# Supplementary material for: Chromothripsis during telomere crisis is independent of NHEJ, and consistent with a replicative origin
Source: Genome Res. 2019 May;29(5):737–49. doi: 10.1101/gr.240705.118 (PMC6499312; doi:10.1101/gr.240705.118)
Supplement: Supplemental Material [file supp_gr.240705.118_Supplemental_file_1.zip › contigs/annotated_contigs/DB53/contig.2.DB53_length_271_mean_cov_13.0553505535.docx]

**DB53_length_271_mean_cov_13.0553505535**

```
GCGTTGCACAGGCCGCCTGCCTGCGCGGGCACCCTGCCAGCTTGTCCCGGGTGCCTTGCTCTTCGATTCTGAAACCAGATCTGAATCCT
 >chr3:75718368-75718578 - E=4e-85
GGACTCCGGGAGGCCCATCTCTCTGGCCGGATCTTCCCTGGCGGCGATGCCTGGAAAGCGATCCTTCTCAAAGGCTCGGAGGAGCAGGG

CGGTCTGGGATCTGGTGACGGCGGTCCGCTTT|TGCGGATGT|AACAAGAGTTACATCTCCTGGGTGATCAGTGTAGAAATATGTCACA
                                          >chrUn_gl000225:87796-87848 - E=6e-14
AAGCCC
```
